# The genome sequence of the Middle-barred Minor moth, *Oligia fasciuncula *(Haworth, 1809)

**DOI:** 10.12688/wellcomeopenres.22744.1

**Published:** 2024-07-22

**Authors:** Gavin R. Broad, Stephanie Holt, Laura Sivess, Natural History Museum Genome Acquisition Lab, Darwin Tree of Life Barcoding collective, Wellcome Sanger Institute Tree of Life Management, Samples and Laboratory team, Wellcome Sanger Institute Scientific Operations: Sequencing Operations, Wellcome Sanger Institute Tree of Life Core Informatics team, Tree of Life Core Informatics collective, Darwin Tree of Life Consortium

**Affiliations:** 1 Natural History Museum, London, England, UK

**Keywords:** Oligia fasciuncula, Middle-barred Minor moth, genome sequence, chromosomal, Lepidoptera

## Abstract

We present a genome assembly from an individual male *Oligia fasciuncula* (the Middle-barred Minor moth; Arthropoda; Insecta; Lepidoptera; Noctuidae). The genome sequence spans 617.70 megabases. Most of the assembly is scaffolded into 31 chromosomal pseudomolecules, including the Z sex chromosome. The mitochondrial genome has also been assembled and is 15.35 kilobases in length. Gene annotation of this assembly on Ensembl identified 17,994 protein-coding genes.

## Species taxonomy

Eukaryota; Opisthokonta; Metazoa; Eumetazoa; Bilateria; Protostomia; Ecdysozoa; Panarthropoda; Arthropoda; Mandibulata; Pancrustacea; Hexapoda; Insecta; Dicondylia; Pterygota; Neoptera; Endopterygota; Amphiesmenoptera; Lepidoptera; Glossata; Neolepidoptera; Heteroneura; Ditrysia; Obtectomera; Noctuoidea; Noctuidae; Noctuinae; Apameini; *Oligia*; *Oligia fasciuncula* (Haworth, 1809) (NCBI:txid1870138).

## Background

*Oligia fasciuncula*, Middle-barred Minor, is straightforward to identify, in contrast with other species of *Oligia,* which usually require genitalia examination for identification. It is a small, compact noctuid moth, reddish- or orange-brown with a darker central stripe, conspicuously bordered with white on either side, towards the inner side of the forewing. Also unlike other *Oligia*, there are no frequent melanic forms of *O. fasciuncula* and the overall variation in colour is rather minor.

*Oligia fasciuncula* is a familiar moth at light traps throughout Britain, favouring damp grassland, including wet woodland ( South, 1907; Waring *et al.*, 2017). The overall picture of the British and Irish population of *O. fasciuncula* is of no overall change in abundance but a small increase in distribution ( Randle *et al.*, 2019). Its range in Europe is fairly limited, mostly western, not occurring far South or North ( GBIF Secretariat, 2024). The larvae feed from late summer, over-winter and resume feeding in the spring, with adults emerging in summer, mainly in June and July. Favoured foodplants are Tufted Hairgrass ( *Deschampsia cespitosa*) and Yorkshire Fog ( *Holcus lanatus*) ( Henwood *et al.*, 2020; South, 1907); larvae have different colour forms and feed on the leaves at night. To our knowledge, there are no published records of parasitoid wasps attacking *O. fasciuncula*, which is more of an indication of how infrequently larvae are reared rather than of its attractiveness to parasitoids.

Here we present a chromosomally complete genome sequence for *Oligia fasciuncula*, based on one male specimen from Selborne, UK.

## Genome sequence report

**Figure 1.**
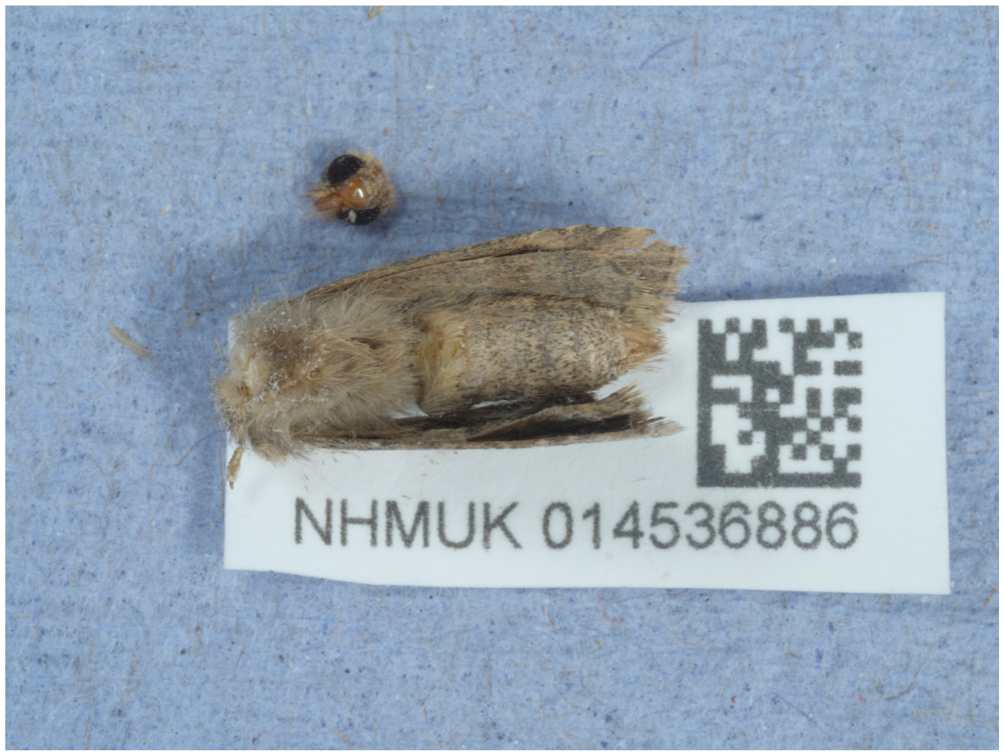
Photograph of the *Oligia fasciuncula* (ilOliFasc1) specimen used for genome sequencing.

The genome of an adult male *Oligia fasciuncula* ( Figure 1) was sequenced using Pacific Biosciences single-molecule HiFi long reads, generating a total of 23.24 Gb (gigabases) from 1.94 million reads, providing approximately 36-fold coverage. Primary assembly contigs were scaffolded with chromosome conformation Hi-C data, which produced 129.50 Gbp from 857.60 million reads, yielding an approximate coverage of 210-fold. Specimen and sequencing information is summarised in Table 1.

**Table 1.** Specimen and sequencing data for *Oligia fasciuncula*.

Project information			
**Study title**	Oligia fasciuncula		
**Umbrella BioProject**	PRJEB63424		
**Species**	*Oligia fasciuncula*		
**BioSample**	SAMEA112221824		
**NCBI taxonomy ID**	1870138		
Specimen information			
Technology	ToLID	BioSample accession	Organism part
**PacBio long read sequencing**	ilOliFasc1	SAMEA112221910	whole_organism
**Hi-C sequencing**	ilOliFasc1	SAMEA112221910	whole_organism
**RNA sequencing**	ilOliFasc2	SAMEA112222374	whole_organism
Sequencing information			
Platform	Run accession	Read count	Base count (Gb)
**Hi-C Illumina NovaSeq 6000**	ERR11606308	8.58e+08	129.5
**PacBio Sequel IIe**	ERR11593796	1.94e+06	23.24
**RNA Illumina NovaSeq X**	ERR12861037	5.92e+07	8.93

**Figure 2.**
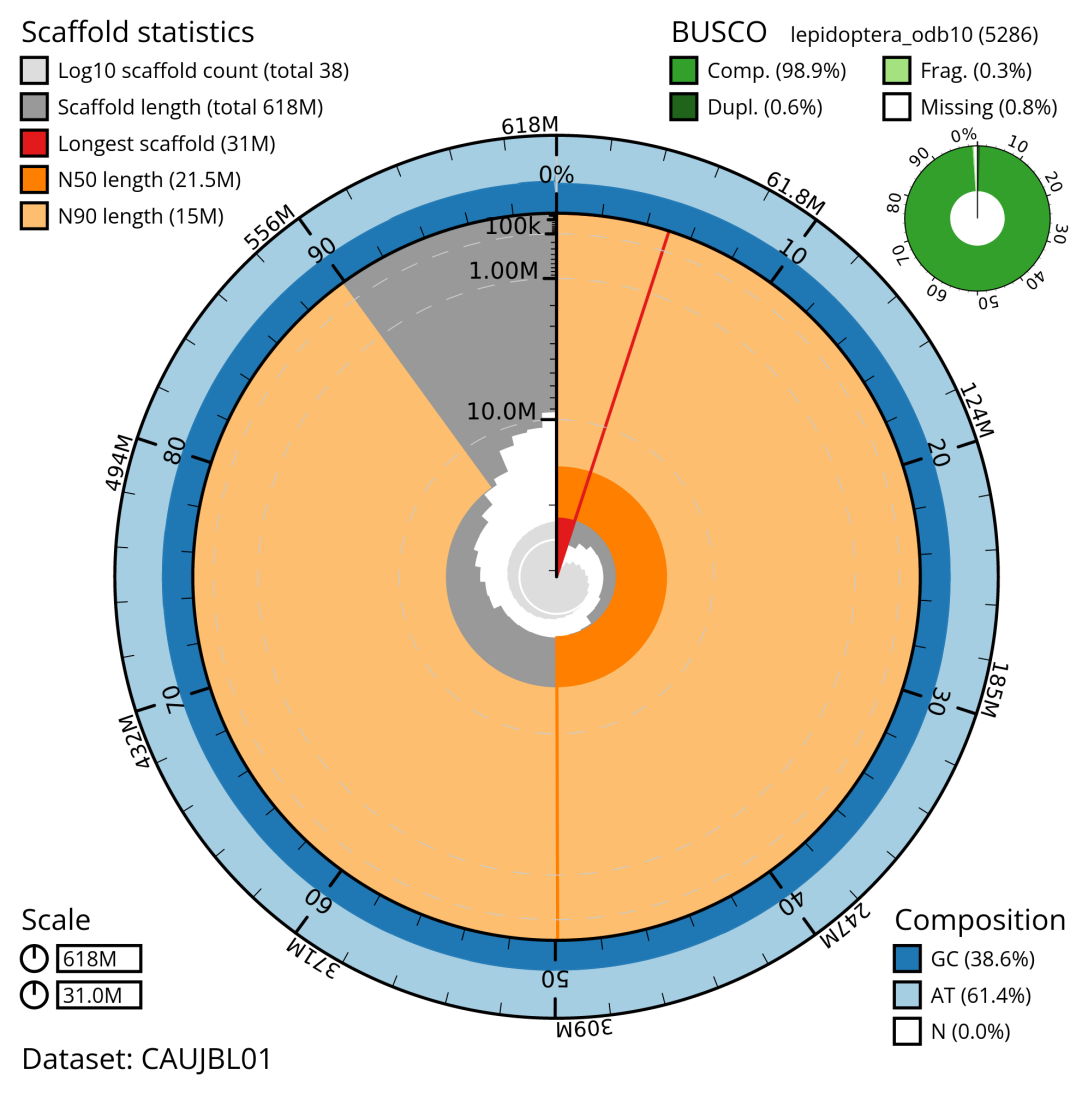
Genome assembly of *Oligia fasciuncula*, ilOliFasc1.1: metrics. The BlobToolKit snail plot shows N50 metrics and BUSCO gene completeness. The main plot is divided into 1,000 size-ordered bins around the circumference with each bin representing 0.1% of the 617,739,502 bp assembly. The distribution of scaffold lengths is shown in dark grey with the plot radius scaled to the longest scaffold present in the assembly (31,038,769 bp, shown in red). . Orange and pale-orange arcs show the N50 and N90 scaffold lengths (21,512,857 and 15,045,205 bp), respectively. The pale grey spiral shows the cumulative scaffold count on a log scale with white scale lines showing successive orders of magnitude. The blue and pale-blue area around the outside of the plot shows the distribution of GC, AT and N percentages in the same bins as the inner plot. A summary of complete, fragmented, duplicated and missing BUSCO genes in the lepidoptera_odb10 set is shown in the top right. An interactive version of this figure is available at https://blobtoolkit.genomehubs.org/view/CAUJBL01/dataset/CAUJBL01/snail.

**Figure 3.**
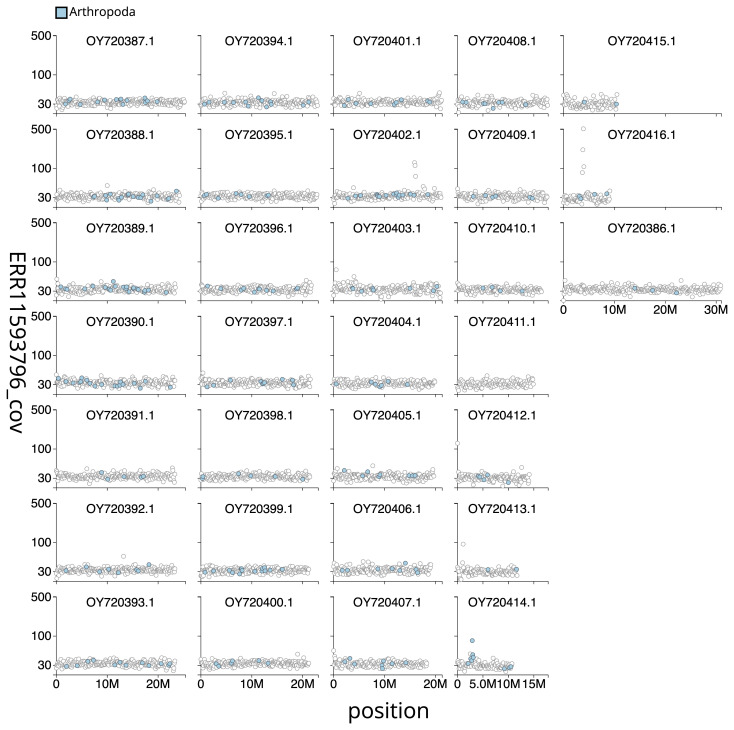
Genome assembly of *Oligia fasciuncula*, ilOliFasc1.1: Distribution plot of base coverage in ERR11593796 against position for sequences in assembly ilOliFasc1.1. Windows of 100 kb are coloured by phylum. The assembly has been filtered to exclude sequences with length < 2,550,000. An interactive version of this figure may be viewed here.

**Figure 4.**
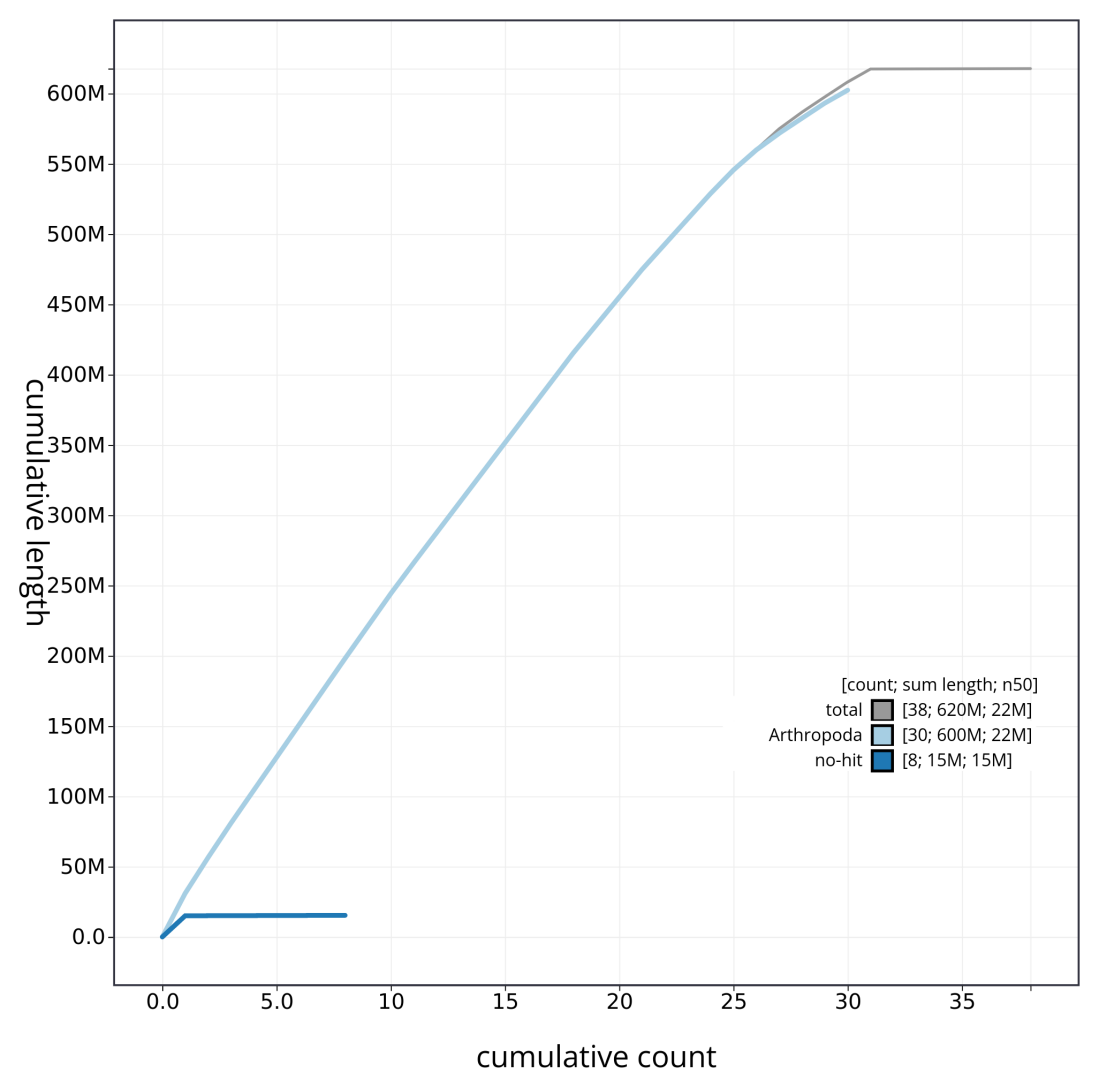
Genome assembly of *Oligia fasciuncula* ilOliFasc1.1: BlobToolKit cumulative sequence plot. The grey line shows cumulative length for all sequences. Coloured lines show cumulative lengths of sequences assigned to each phylum using the buscogenes taxrule. An interactive version of this figure is available at https://blobtoolkit.genomehubs.org/view/CAUJBL01/dataset/CAUJBL01/cumulative.

**Figure 5.**
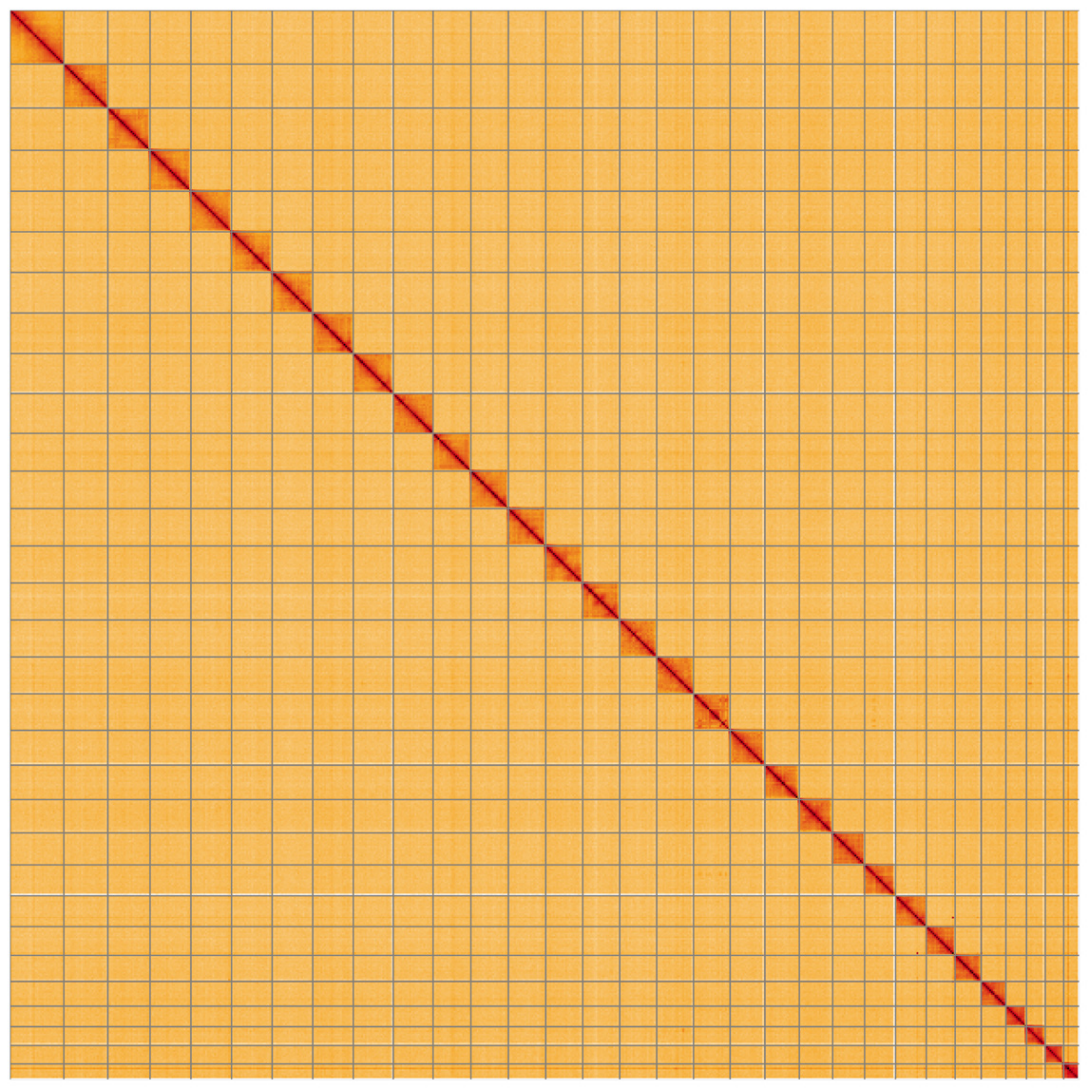
Genome assembly of *Oligia fasciuncula* ilOliFasc1.1: Hi-C contact map of the ilOliFasc1.1 assembly, visualised using HiGlass. Chromosomes are shown in order of size from left to right and top to bottom. An interactive version of this figure may be viewed at https://genome-note-higlass.tol.sanger.ac.uk/l/?d=RayLJUdLR6GB20MCpfvelg.

Manual assembly curation corrected 6 missing joins or mis-joins and 2 haplotypic duplications, reducing the scaffold number by 2.56%, and increasing the scaffold N50 by 0.63%. The final assembly has a total length of 617.70 Mb in 37 sequence scaffolds with a scaffold N50 of 21.5 Mb ( Table 2), with 60 gaps. The snail plot in Figure 2 provides a summary of the assembly statistics, while Figure 3 shows the distribution of assembly scaffolds based on base coverage across chromosomes. The cumulative assembly plot in Figure 4 shows curves for subsets of scaffolds assigned to different phyla. Most (99.95%) of the assembly sequence was assigned to 31 chromosomal-level scaffolds, representing 30 autosomes and the Z sex chromosome. Chromosome-scale scaffolds confirmed by the Hi-C data are named in order of size ( Figure 5; Table 3). The Z chromosome was assigned based on synteny to *Oligia strigilis* (GCA_951800025.1) ( Broad *et al.*, 2024) and *Oligia latruncula* (GCA_948474745.1) ( Broad *et al.*, 2023). While not fully phased, the assembly deposited is of one haplotype. Contigs corresponding to the second haplotype have also been deposited. The mitochondrial genome was also assembled and can be found as a contig within the multifasta file of the genome submission.

**Table 2.** Genome assembly data for *Oligia fasciuncula*, ilOliFasc1.1.

Genome assembly		
Assembly name	ilOliFasc1.1	
Assembly accession	GCA_963082905.1	
*Accession of alternate haplotype*	*GCA_963082925.1*	
Span (Mb)	617.70	
Number of contigs	98	
Contig N50 length (Mb)	11.9	
Number of scaffolds	37	
Scaffold N50 length (Mb)	21.5	
Longest scaffold (Mb)	31.04	
Assembly metrics *		*Benchmark*
Consensus quality (QV)	66.0	*≥ 50*
*k*-mer completeness	100.0%	*≥ 95%*
BUSCO **	C:98.9%[S:98.3%,D:0.6%],F:0.3%,M:0.8%,n:5,286	*C ≥ 95%*
Percentage of assembly mapped to chromosomes	99.95%	*≥ 95%*
Sex chromosomes	Z	*localised homologous pairs*
Organelles	Mitochondrial genome: 15.35 kb	*complete single alleles*
Genome annotation of assembly GCA_963082905.1 at Ensembl		
Number of protein-coding genes	17,994	
Number of gene transcripts	18,208	

* Assembly metric benchmarks are adapted from column VGP-2020 of “Table 1: Proposed standards and metrics for defining genome assembly quality” from Rhie *et al.* (2021). ** BUSCO scores based on the lepidoptera_odb10 BUSCO set using version 5.3.2. C = complete [S = single copy, D = duplicated], F = fragmented, M = missing, n = number of orthologues in comparison. A full set of BUSCO scores is available at https://blobtoolkit.genomehubs.org/view/Oligia%20fasciuncula/dataset/CAUJBL01/busco.

**Table 3.** Chromosomal pseudomolecules in the genome assembly of *Oligia fasciuncula*, ilOliFasc1.

INSDC accession	Name	Length (Mb)	GC%
OY720387.1	1	25.4	38.5
OY720388.1	2	24.39	38.5
OY720389.1	3	23.59	38.5
OY720390.1	4	23.49	38.5
OY720391.1	5	23.43	38.5
OY720392.1	6	23.41	38.0
OY720393.1	7	23.4	38.5
OY720394.1	8	23.06	38.0
OY720395.1	9	23.0	38.5
OY720396.1	10	21.8	38.5
OY720397.1	11	21.67	38.0
OY720398.1	12	21.51	38.0
OY720399.1	13	21.39	38.5
OY720400.1	14	21.38	38.0
OY720401.1	15	21.33	38.5
OY720402.1	16	21.25	39.0
OY720403.1	17	21.13	38.5
OY720404.1	18	20.07	38.5
OY720405.1	19	19.9	38.5
OY720406.1	20	19.23	39.0
OY720407.1	21	18.46	38.5
OY720408.1	22	17.82	39.0
OY720409.1	23	17.78	39.0
OY720410.1	24	16.63	39.0
OY720411.1	25	15.05	39.0
OY720412.1	26	14.29	39.0
OY720413.1	27	11.83	39.5
OY720414.1	28	10.88	39.5
OY720415.1	29	10.63	40.0
OY720416.1	30	9.21	41.0
OY720386.1	Z	31.04	38.0
OY720417.1	MT	0.02	20.0

The estimated Quality Value (QV) of the final assembly is 66.0 with *k*-mer completeness of 100.0%, and the assembly has a BUSCO v5.3.2 completeness of 98.9% (single = 98.3%, duplicated = 0.6%), using the lepidoptera_odb10 reference set ( *n* = 5,286).

Metadata for specimens, BOLD barcode results, spectra estimates, sequencing runs, contaminants and pre-curation assembly statistics are given at https://links.tol.sanger.ac.uk/species/1870138.

## Genome annotation report

The *Oligia fasciuncula* genome assembly (GCA_963082905.1) was annotated at the European Bioinformatics Institute (EBI) on Ensembl Rapid Release. The resulting annotation includes 18,208 transcribed mRNAs from 17,994 protein-coding genes ( Table 2; https://rapid.ensembl.org/Oligia_fasciuncula_GCA_963082905.1/Info/Index). The average transcript length is 8,259.94. There are 1.01 coding transcripts per gene and 5.53 exons per transcript.

## Methods

### Sample acquisition

Adult *Oligia fasciuncula* specimens were collected from Gilbert White's House, Selborne, England, UK (latitude 51.09, longitude –0.94) on 2021-06-10, using a light trap. The collectors were Stephanie Holt, Gavin Broad and Laura Sivess (Natural History Museum). The specimens were formally identified by Gavin Broad (Natural History Museum), and then preserved by dry freezing at –80 °C. Specimen NHMUK014536886 (ToLID ilOliFasc1) was used for genome sequencing and Hi-C scaffolding, and specimen NHMUK014536847 (ToLID ilOliFasc2) was used for RNA sequencing.

In addition to identification based on morphology, the species taxonomy was verified by DNA barcoding soon after collection, according to the framework developed by Twyford *et al.* (2024). A small sample was dissected from the specimens and stored in ethanol. The tissue was lysed, and the COI marker region was amplified by PCR. Amplicons were sequenced and compared to the BOLD database, confirming the species identification ( Crowley *et al.*, 2023). The standard operating procedures for the Darwin Tree of Life barcoding have been deposited on protocols.io ( Beasley *et al.*, 2023). The remaining parts of the specimen were shipped on dry ice to the Wellcome Sanger Institute (WSI). A DNA barcode was also generated from the PacBio sequencing data at a later stage for sample tracking through the genome production pipeline at the WSI ( Twyford *et al.*, 2024).

### Nucleic acid extraction

The workflow for high molecular weight (HMW) DNA extraction at the Wellcome Sanger Institute (WSI) Tree of Life Core Laboratory includes a sequence of core procedures: sample preparation; sample homogenisation, DNA extraction, fragmentation, and clean-up. In sample preparation, the ilOliFasc1 sample was weighed and dissected on dry ice, setting aside tissue for Hi-C sequencing ( Jay *et al.*, 2023). Tissue from the whole organism was homogenised using a PowerMasher II tissue disruptor ( Denton *et al.*, 2023a).

HMW DNA was extracted in the WSI Scientific Operations core using the Automated MagAttract v2 protocol ( Oatley *et al.*, 2023). The DNA was sheared into an average fragment size of 12–20 kb in a Megaruptor 3 system with speed setting 31 ( Bates *et al.*, 2023). Sheared DNA was purified by solid-phase reversible immobilisation ( Strickland *et al.*, 2023): in brief, the method employs a 1.8X ratio of AMPure PB beads to sample to eliminate shorter fragments and concentrate the DNA. The concentration of the sheared and purified DNA was assessed using a Nanodrop spectrophotometer and Qubit Fluorometer using the Qubit dsDNA High Sensitivity Assay kit. Fragment size distribution was evaluated by running the sample on the FemtoPulse system.

RNA was extracted from tissue of the whole organism of ilOliFasc2 in the Tree of Life Laboratory at the WSI using the RNA Extraction: Automated MagMax™ *mir*Vana protocol ( do Amaral *et al.*, 2023). The RNA concentration was assessed using a Nanodrop spectrophotometer and a Qubit Fluorometer using the Qubit RNA Broad-Range Assay kit. Analysis of the integrity of the RNA was done using the Agilent RNA 6000 Pico Kit and Eukaryotic Total RNA assay.

Protocols developed by the WSI Tree of Life laboratory are publicly available on protocols.io ( Denton *et al.*, 2023b).

### Sequencing

Pacific Biosciences HiFi circular consensus DNA sequencing libraries were constructed according to the manufacturers’ instructions. Poly(A) RNA-Seq libraries were constructed using the NEB Ultra II RNA Library Prep kit. DNA and RNA sequencing was performed by the Scientific Operations core at the WSI on Pacific Biosciences Sequel IIe (HiFi) and Illumina NovaSeq X (RNA-Seq) instruments. Hi-C data were also generated from remaining tissue of ilOliFasc1 using the Arima-HiC v2 kit. The Hi-C sequencing was performed using paired-end sequencing with a read length of 150 bp on the Illumina NovaSeq 6000 instrument.

### Genome assembly, curation and evaluation

***Assembly***

The original assembly of HiFi reads was performed using Hifiasm ( Cheng *et al.*, 2021) with the --primary option. Haplotypic duplications were identified and removed with purge_dups ( Guan *et al.*, 2020). Hi-C reads are further mapped with bwa-mem2 ( Vasimuddin *et al.*, 2019) to the primary contigs, which are further scaffolded using the provided Hi-C data ( Rao *et al.*, 2014) in YaHS ( Zhou *et al.*, 2023) using the --break option. Scaffolded assemblies are evaluated using Gfastats ( Formenti *et al.*, 2022), BUSCO ( Manni *et al.*, 2021) and MERQURY.FK ( Rhie *et al.*, 2020).

The mitochondrial genome was assembled using MitoHiFi ( Uliano-Silva *et al.*, 2023), which runs MitoFinder ( Allio *et al.*, 2020) and uses these annotations to select the final mitochondrial contig and to ensure the general quality of the sequence.

***Assembly curation***

The assembly was decontaminated using the Assembly Screen for Cobionts and Contaminants (ASCC) pipeline (article in preparation). Manual curation was primarily conducted using PretextView ( Harry, 2022), with additional insights provided by JBrowse2 ( Diesh *et al.*, 2023) and HiGlass ( Kerpedjiev *et al.*, 2018). Scaffolds were visually inspected and corrected as described by Howe *et al.* (2021). Any identified contamination, missed joins, and mis-joins were corrected, and duplicate sequences were tagged and removed. Sex chromosomes were identified by synteny. The entire process is documented at https://gitlab.com/wtsi-grit/rapid-curation (article in preparation).

***Evaluation of the final assembly***

A Hi-C map for the final assembly was produced using bwa-mem2 ( Vasimuddin *et al.*, 2019) in the Cooler file format ( Abdennur & Mirny, 2020). To assess the assembly metrics, the *k*-mer completeness and QV consensus quality values were calculated in Merqury ( Rhie *et al.*, 2020). This work was done using Nextflow ( Di Tommaso *et al.*, 2017) DSL2 pipelines “sanger-tol/readmapping” ( Surana *et al.*, 2023a) and “sanger-tol/genomenote” ( Surana *et al.*, 2023b). The genome was analysed within the BlobToolKit environment ( Challis *et al.*, 2020) and BUSCO scores ( Manni *et al.*, 2021; Simão *et al.*, 2015) were calculated.

Table 4 contains a list of relevant software tool versions and sources.

**Table 4.** Software tools: versions and sources.

Software tool	Version	Source
BlobToolKit	4.2.1	https://github.com/blobtoolkit/blobtoolkit
BUSCO	5.3.2	https://gitlab.com/ezlab/busco
Hifiasm	0.16.1-r375	https://github.com/chhylp123/hifiasm
HiGlass	1.11.6	https://github.com/higlass/higlass
Merqury	MerquryFK	https://github.com/thegenemyers/MERQURY.FK
MitoHiFi	2	https://github.com/marcelauliano/MitoHiFi
PretextView	0.2	https://github.com/wtsi-hpag/PretextView
purge_dups	1.2.3	https://github.com/dfguan/purge_dups
sanger-tol/genomenote	v1.0	https://github.com/sanger-tol/genomenote
sanger-tol/readmapping	1.1.0	https://github.com/sanger-tol/readmapping/tree/1.1.0
YaHS	yahs-1.1.91eebc2	https://github.com/c-zhou/yahs

### Genome annotation

The BRAKER2 pipeline ( Brůna *et al.*, 2021) was used in the default protein mode to generate annotation for the *Oligia fasciuncula* assembly (GCA_963082905.1) in Ensembl Rapid Release at the EBI.

### Wellcome Sanger Institute – Legal and Governance

The materials that have contributed to this genome note have been supplied by a Darwin Tree of Life Partner. The submission of materials by a Darwin Tree of Life Partner is subject to the **‘Darwin Tree of Life Project Sampling Code of Practice’**, which can be found in full on the Darwin Tree of Life website here. By agreeing with and signing up to the Sampling Code of Practice, the Darwin Tree of Life Partner agrees they will meet the legal and ethical requirements and standards set out within this document in respect of all samples acquired for, and supplied to, the Darwin Tree of Life Project.

Further, the Wellcome Sanger Institute employs a process whereby due diligence is carried out proportionate to the nature of the materials themselves, and the circumstances under which they have been/are to be collected and provided for use. The purpose of this is to address and mitigate any potential legal and/or ethical implications of receipt and use of the materials as part of the research project, and to ensure that in doing so we align with best practice wherever possible. The overarching areas of consideration are:

●   Ethical review of provenance and sourcing of the material

●   Legality of collection, transfer and use (national and international)

Each transfer of samples is further undertaken according to a Research Collaboration Agreement or Material Transfer Agreement entered into by the Darwin Tree of Life Partner, Genome Research Limited (operating as the Wellcome Sanger Institute), and in some circumstances other Darwin Tree of Life collaborators.

## Data Availability

European Nucleotide Archive: *Oligia fasciun*cula. Accession number PRJEB63424; https://identifiers.org/ena.embl/PRJEB63424 ( Wellcome Sanger Institute, 2023). The genome sequence is released openly for reuse. The *Oligia fasciuncula* genome sequencing initiative is part of the Darwin Tree of Life (DToL) project. All raw sequence data and the assembly have been deposited in INSDC databases. Raw data and assembly accession identifiers are reported in Table 1 and Table 2.

## Author information

Members of the Natural History Museum Genome Acquisition Lab are listed here: https://doi.org/10.5281/zenodo.12159242.

Members of the Darwin Tree of Life Barcoding collective are listed here: https://doi.org/10.5281/zenodo.4893703.

Members of the Wellcome Sanger Institute Tree of Life Management, Samples and Laboratory team are listed here: https://doi.org/10.5281/zenodo.12162482.

Members of Wellcome Sanger Institute Scientific Operations: Sequencing Operations are listed here: https://doi.org/10.5281/zenodo.12165051.

Members of the Wellcome Sanger Institute Tree of Life Core Informatics team are listed here: https://doi.org/10.5281/zenodo.12160324.

Members of the Tree of Life Core Informatics collective are listed here: https://doi.org/10.5281/zenodo.12205391.

Members of the Darwin Tree of Life Consortium are listed here: https://doi.org/10.5281/zenodo.4783558.
