# Supplementary material for: The genome sequence of the Middle-barred Minor moth, Oligia fasciuncula (Haworth, 1809)
Source: Wellcome Open Res. 2024 Jul 22;9:395. [Version 1] doi: 10.12688/wellcomeopenres.22744.1 (PMC12511861; doi:10.12688/wellcomeopenres.22744.1)
Supplement: Peer review history

# Reviewer report: Reviewer response for version 1

**DOI:** 10.21956/wellcomeopenres.25047.r134153

**Published:** 2025-10-29

**Authors:** Somjit Homchan

**Affiliations:** 1 Department of biology, Naresuan University, Tha Pho, Phitsanulok, Thailand

The chromosomally complete genome assembly of the Middle-barred Minor moth ( *Oligia fasciuncula*) presented in this study provides valuable data for future research. The authors employed several established tools for genome analysis following standard criteria. However, additional information or references clarifying how the 31 chromosomal pseudomolecules were assigned would strengthen the study. It would also be beneficial to include references confirming the physical chromosome number of this species or closely related species to validate the chromosomal scaffolds.

Are sufficient details of methods and materials provided to allow replication by others?

Yes

Is the rationale for creating the dataset(s) clearly described?

Yes

Are the datasets clearly presented in a useable and accessible format?

Yes

Are the protocols appropriate and is the work technically sound?

Yes

Reviewer Expertise:

Molecular markers, Cytogenetics, Population Genetics

I confirm that I have read this submission and believe that I have an appropriate level of expertise to confirm that it is of an acceptable scientific standard.

# Reviewer report: Reviewer response for version 1

**DOI:** 10.21956/wellcomeopenres.25047.r134160

**Published:** 2025-10-09

**Authors:** Shangzhe Zhang

**Affiliations:** 1 University of St Andrews, St Andrews, Scotland, UK

The authors provided a high-quality genome assembly for *Oligia fasciuncula , along with its* annotation. I think the workflow, results and resources are in good format to be indexed. I only have one concern: 

 The authors didn't specify the tools they used in the identification of the Z chromosome, and it would be nice to have an identical alignment proportion from the sex chromosome synteny--though I expect a high value, as reference genomes are from the same genus.

Are sufficient details of methods and materials provided to allow replication by others?

Yes

Is the rationale for creating the dataset(s) clearly described?

Yes

Are the datasets clearly presented in a useable and accessible format?

Yes

Are the protocols appropriate and is the work technically sound?

Yes

Reviewer Expertise:

Genomics; Evolutionary biology; Genomic structural variation.

I confirm that I have read this submission and believe that I have an appropriate level of expertise to confirm that it is of an acceptable scientific standard.

# Reviewer report: Reviewer response for version 1

**DOI:** 10.21956/wellcomeopenres.25047.r90950

**Published:** 2024-08-12

**Authors:** Sophie Tandonnet

**Affiliations:** 1 Universitat de Barcelona, Barcelona, Catalonia, Spain

**Summary:**

 As far as I can tell, this is a beautiful genome assembly from 1 individual *O. fasciuncula*, highly continuous and highly complete in terms of BUSCO. The assembly is based on PacbioHiFi and HiC Arima data. The mitochondrial genome was identified and annotated. The putative Z chromosome was identified through synteny. A second individual was used for RNA-seq and genome annotation. The genome annotation is available. The paper is well written and easy to follow. The protocols used are detailed on the protocols.io and well referenced.  

 The annotation only uses 1 adult individual (of unknown sex) so some genes (only expressed at another stage, in another context or by the other sex) may be missing from the annotation, mis-annotated or missing some isoforms.

 My suggestions for improvement are minor.

**Genome sequence report**

 In this section, the authors summarize the genome sequencing acquired and the final genome metrics (size, N50, number of Gaps, GC content…).

 Fig 4: The figure legend states that there was an interactive version, but it seems static ( https://blobtoolkit.genomehubs.org/view/CAUJBL01/dataset/CAUJBL01/cumulative ).

 "Most (99.95%) of the assembly sequence was assigned to 31 chromosomal-level scaffolds, representing 30 autosomes and the Z sex chromosome"Could the authors add a reference (or justification) supporting the information that the chromosome number for this species is 31?

 "The identity of the Z chromosome was assigned by synteny to the Z chromosome of *Oligia strigilis* (GCA_951800025.1) ( Broad et al. , 2024 ) and *Oligia latruncula* (GCA_948474745.1) ( Broad et al. , 2023 )." I looked at how the Z chromosome assignment was made in O. *strigilis* (GCA_951800025.1) ( Broad et al. , 2024 ) and *Oligia latruncula* (GCA_948474745.1) ( Broad et al. , 2023 ). In both cases the Z chromosome assignment was based on synteny. In *O. strigilis*, the Z chromosome was identified based on synteny with *Oligia latruncula*. The Z chromosome of *Oligia latruncula* was identified through synteny with the Z chromosome of *Apamea epomidion* (GCA_947507525.1; ilApaEpom1). I couldn't find how the Z chromosome os A. *epomidion *was assigned its identity. As this is a chain of synteny-based assignments and the Z chromosome sequence was not demonstrated through differential coverage, qPCR or genetic studies, I suggest the authors write "the putative identity of the Z chromosome…" 

**Methods**

***Nucleic acid extraction***

 The text mentions "In sample preparation, the ilOliFasc1 sample was weighed and dissected on dry ice, setting aside tissue for Hi-C sequencing ( Jay et al. , 2023 )."→Consider adding which part of the specimen was used for PacBio HiFi and for HiC. Also this sentence is at odds with table 1 which states that the "whole organism" was used for HiC and Pacbio.

 It is also not clear how the samples were prepared for Hi-C sequencing. Please consider adding these details.

***Genome assembly, curation and evaluation***

 1. I suggest reworking the following sentence (although this is up to the authors), especially changing the tense to past (to match the rest of the article) and remove redundant words: 

- Original: "Hi-C reads **are** **further** mapped with bwa-mem2 ( Vasimuddin et al. , 2019 ) to the primary contigs, which **are** **further** scaffolded using the provided Hi-C data ( Rao et al. , 2014 ) in YaHS ( Zhou et al. , 2023 ) using the --break option. "

 

- One suggestion: "Hi-C reads **were** mapped with bwa-mem2 ( Vasimuddin et al. , 2019 ) to the primary contigs. The resulting Hi-C reads alignment was then used to scaffold the contigs ( Rao et al. , 2014 ) in YaHS ( Zhou et al. , 2023 ) using the --break option."

2. "the Assembly Screen for Cobionts and Contaminants (ASCC) pipeline (article in preparation)." → Add the Github (or alternative repository) page if available.

 3. "Sex chromosomes were identified by synteny." → Give more detail on how this was done, unless this is part of the rapid curation pipeline.

Are sufficient details of methods and materials provided to allow replication by others?

Yes

Is the rationale for creating the dataset(s) clearly described?

Yes

Are the datasets clearly presented in a useable and accessible format?

Yes

Are the protocols appropriate and is the work technically sound?

Yes

Reviewer Expertise:

Evolutionary biology, Sex determination, Genomics

I confirm that I have read this submission and believe that I have an appropriate level of expertise to confirm that it is of an acceptable scientific standard.
